# Epitaxial Growth and Characterization of AlInN-Based Core-Shell Nanowire Light Emitting Diodes Operating in the Ultraviolet Spectrum

**DOI:** 10.1038/s41598-020-59442-0

**Published:** 2020-02-13

**Authors:** Ravi Teja Velpula, Barsha Jain, Moab Rajan Philip, Hoang Duy Nguyen, Renjie Wang, Hieu Pham Trung Nguyen

**Affiliations:** 1 Department of Electrical and Computer Engineering, New Jersey Institute of Technology, 323 Dr Martin Luther King Jr Boulevard, Newark, New Jersey 07102 United States; 2 Institute of Chemical Technology, Vietnam Academy of Science and Technology, 1 Mac Dinh Chi Street, District 1, Ho Chi Minh City, 700000 Vietnam; 3 Department of Engineering Physics, McMaster University, 1280 Main Street West, Hamilton, Ontario, L8S 4L7 Canada

**Keywords:** Nanowires, Inorganic LEDs

## Abstract

We report the demonstration of the first axial AlInN ultraviolet core-shell nanowire light-emitting diodes with highly stable emission in the ultraviolet wavelength range. During epitaxial growth of the AlInN layer, an AlInN shell is spontaneously formed, resulting in reduced nonradiative recombination on the nanowire surface. The AlInN nanowires exhibit a high internal quantum efficiency of ~52% at room temperature for emission at 295 nm. The peak emission wavelength can be varied from 290 nm to 355 nm by changing the growth conditions. Moreover, significantly strong transverse magnetic (TM) polarized emission is recorded, which is ~4 times stronger than the transverse electric (TE) polarized light at 295 nm. This study provides an alternative approach for the fabrication of new types of high-performance ultraviolet light emitters.

## Introduction

A compact, highly efficient, and high-power ultraviolet (UV) light source with emission wavelengths below 350 nm has been intensively investigated for several emerging applications. UV light emitters have primarily been used in several key applications, including remote detection of biological and chemical compounds^1^, phototherapy^2^, water/air/surface purification and disinfection^3,4^, cancer detection^5^ and fluorescence sensing or Raman spectroscopy^6^. Currently, major UV light-emitting diode (LED) customers are users of UV lights in the wavelength range from 280 nm to 400 nm, representing more than 90% of the total UV light-source market^7^. Among these applications, UV curing is the most dynamic and most important market due to significant advantages offered over traditional technologies, including lower cost of ownership, system miniaturization, etc.^7^. AlGaN-based UV LEDs have been widely developed for deep-UV emission, with wavelengths shorter than 280 nm. Nevertheless, the performance of AlGaN-based deep-UV LEDs remains very poor, which has been affected by several factors that may include the high density of dislocations and the poor *p-*type doping, resulting in the low output power^7–10^. Moreover, the device light extraction efficiency (LEE) is further limited by UV light polarization, particularly in the ~290–355 nm wavelength region, when the UV light polarization changes from transverse electric (TE) to transverse magnetic (TM) polarization due to prohibitively high Al-composition and nanowire geometry^11,12^. As such, the polarization state switches from TE to TM, reducing the LEE of the related UV LEDs^13^. Because of these barriers, the external quantum efficiency (EQE) of the UV LEDs with emission wavelengths above 300 nm can reach up to nearly 10%. However, their performance deteriorates drastically with decreasing wavelengths^14^. For instance, the EQE of UV LEDs with peak wavelengths below 250 nm decreases dramatically from less than 1% to ~0.04% and has an extremely low output power, which is just a few tens of nW for emission 210 nm^8^. Such power is extremely low for practical applications.

Until recently, fundamental and applied research approaches for light emitters have essentially focused on InGaN and AlGaN alloys for near-UV^15,16^ and UV photonic devices, respectively, while the approach of utilizing alternative group III nitride UV materials has not been reported. In this regard, the Al_x_In_1-x_N alloy has not been widely studied, even though it holds great potential application in UV and visible light-emitting devices. For example, a lattice match could be achieved when AlInN with an In composition of ~17–18% was grown on GaN^17,18^, and the large contrast of the refractive index to GaN makes these a highly promising candidate for UV light emitters. Recent studies have shown that AlInN offers a large optical gain for deep-UV LEDs^19^. Free of defects and quantum-confined Stark effects were achieved for *m*-plan GaN/AlInN core-shell nanowire UV emitters^20^. Using k.p perturbation theory, Fu *et al*. reported that AlInN compounds can be grown on both GaN and AlN templates, while AlGaN is detrimental to growth on GaN templates^21^. AlInN offers wider windows of optimal alloy composition for UV emission than AlGaN, especially to deep-UV emission^21^. AlInN provides several advantages and is of great interest for replacing AlGaN or InGaN in several photonic and electronic devices. For instance, lattice matched GaN/AlInN superlattices have been utilized for intersubband transitions^22^, high-reflectivity distributed Bragg reflectors^23^, high-quality microcavities for vertical cavity surface emitting laser structures^24^, and the realization of high-performance high-electron-mobility transistors^25^.

Despite tremendous advantages, AlInN semiconductor research is highly limited due to the immature epitaxial growth of high-quality AlInN. Molecular beam epitaxy (MBE) growth of group-III nitrides under metal-rich conditions usually provides smooth surface morphologies at low growth temperatures. However, nitrogen-rich growth at these low temperatures results in rough surfaces^26^. The main growth issue for AlInN by MBE is composition inhomogeneity, which is commonly presented in the AlInN layer^27,28^. The difficulties in epitaxial growth of AlInN have resulted from extremely large differences in optimal growth temperatures for InN (~450 °C) and AlN (~800 °C)^29^. Additionally, inefficient *p-*type doping in AlInN also strongly affects the electrical properties of the related devices. Such difficulties result in low crystalline quality and low device performance. The MBE growth under nitrogen-rich conditions offers an effective approach to eliminate the composition inhomogeneity in the AlInN, as reported by Speck *et al*.^30,31^. By decreasing the Al flux and growth of AlInN under N-rich conditions, homogenous AlInN layers with high In contents could be achieved^30,31^. Therefore, nitrogen-rich grown nanowires seem to be the best option offering homogeneous AlInN structures nearly free of dislocations at high In contents. However, to the best of our knowledge, axial nanowire-based AlInN semiconductors grown by MBE have not been reported, even though nanowire structures offer several advantages. Nanowire structures have significant attributes, for instance, significantly improved light output power due to significantly reduced dislocations and polarization fields^32,33^. High-performance group III-nitride nanowire LEDs have been successfully achieved on Si substrates^32,34,35^. Moreover, the surface doping and electrical conductivity of nanowire LEDs can be improved due to the reduced formation energy of the substitutional doping on the near surface area^36^.

In this paper, we conducted a detailed investigation of the epitaxial growth and structural and optical characteristics of Al_x_In_1-x_N/GaN nanowires on Si (111) substrate by plasma-assisted MBE (PAMBE). We have also further demonstrated the first axial AlInN core-shell nanowire UV LED heterostructures operating in the UV-A and UV-B bands. An AlInN shell is spontaneously formed during the growth of the AlInN epilayer, resulting in significantly decreased nonradiative recombination on the nanowire surfaces. The peak wavelength can be tuned from 290 nm to 355 nm by altering the aluminium content in the AlInN active region. The AlInN UV nanowires with an emission wavelength of 295 nm exhibit a relatively high internal quantum efficiency (IQE) of ~52%. Moreover, the UV LED device exhibits strong UV light emission with highly stable peak emission at 295 nm. The polarized optical properties of AlInN nanowire LEDs were also investigated. It is suggested that the UV light from AlInN nanowire LEDs is mainly TM polarized with emission approximately 4 times stronger than that of TE light.

## Results

### Structural characterizations

**Figure 1:**
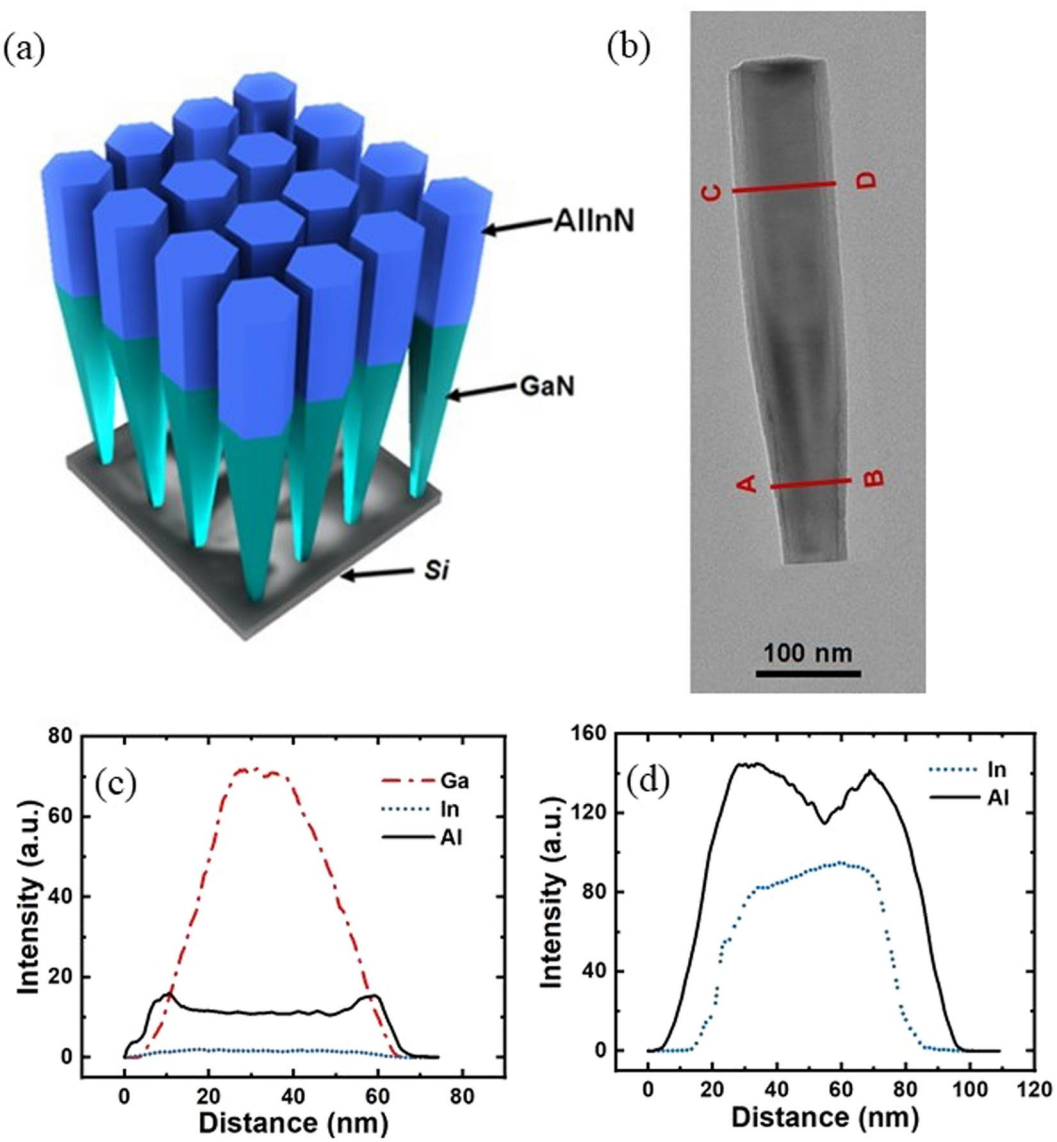
(**a**) Schematic structure of the AlInN nanowire on the GaN template. (**b**) TEM image of the AlInN/GaN nanowire in which the presence of a core-shell structure is clearly shown. EDXS line scan profile showing the quantitative variation in Ga, In and Al signals along lines 1–2. (**c**) and variation in the In and Al signal along lines 3–4.

In this study, AlInN nanowire light emitters were grown on *n*-Si (111) substrates by a customized Veeco Gen II MBE system. As illustrated in Fig. 1(a), the GaN nanowire template was first grown on Si to facilitate the formation of the AlInN segment. Subsequently, a detailed study of the epitaxial growth of AlInN nanowires on GaN nanowire templates was performed to acquire the optimal growth conditions for AlInN nanowire LEDs. The structural properties of AlInN nanowires were characterized by scanning transmission electron microscopy (STEM). Figure 1(b) confirms the presence of GaN and AlInN segments. The wire diameter increased from the GaN segment to the AlInN portion and remained constant at the top of the nanowire, similar to our reported studies on InGaN and AlGaN nanowires^35,37,38^. Moreover, it was also suggested that a core-shell AlInN/GaN structure spontaneously formed during the epitaxial growth of the AlInN layer. The compositional distribution in the nanowire can be characterized by energy dispersive X-ray spectrometry (EDXS) analysis, which was performed in the GaN and AlInN regions, indicated as lines 1–2 and 3–4 in Fig. 1(b), respectively. As presented in Fig. 1(c), corresponding to line scans 1–2, the Ga signal showed the highest intensity at the core of the nanowire and decreased at the sidewalls of the nanowire. However, the Al signal reached its peaks at the sidewalls and decreased towards the nanowire centre. The presence of In was also determined by the detected In signals, even though the In signal was significantly lower than the Ga and Al signals. Therefore, it was suggested that a unique GaN/AlInN radial core-shell heterostructure was grown. At the top portion of the nanowire corresponding to line scans 3–4, an AlInN shell around the AlInN core was also evidenced by the EDXS line scan, as shown in Fig. 1(d). The In signal was localized inside the nanowire. The Al signal was again maximal at the sidewalls and significantly reduced in the core section of the wire. The thickness of the shell layer was estimated to be approximately 13.6 nm at the nanowire top and gradually decreased to approximately 8.4 nm at the nanowire bottom. The formation of these core-shell nanowire structures is similar to what we have reported for AlGaN/GaN and AlGaN/AlInN core-shell nanowires^39,40^. Moreover, the presence of the shell layer may significantly improve the optical properties of the underlying GaN nanowire templates and the AlInN core, which is similar to those reported for AlInGaN/GaN^41^, AlGaN/GaN^38,42–44^, and AlGaN/InGaN^39,40^ core-shell nanowire structures.

**Figure 2:**
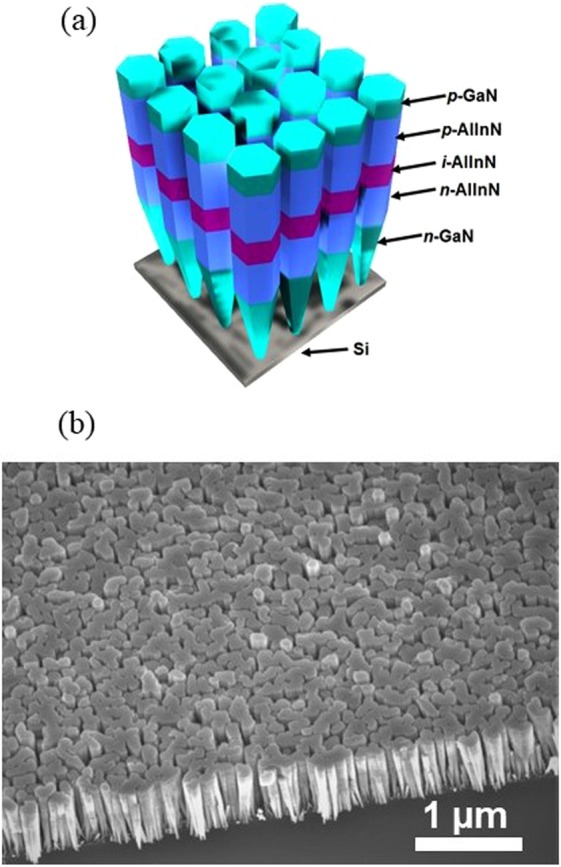
(**a**) Schematic illustration of the AlInN nanowire LED structure on Si. (**b**) 45° tilted scanning electron microscopy image of a typical AlInN nanowire LED sample showing uniform nanowires on Si.

We further developed AlInN/GaN UV nanowire LEDs on Si substrates utilizing the optimal growth conditions of AlInN nanowires on GaN templates. The schematic structure of the device is illustrated in Fig. 2(a), which includes an ~200 nm GaN:Si segment, a 100 nm Al_x_In_1-x_N:Si/40 nm *i-*Al_y_In_1-y_N/100 nm Al_x_In_1-x_N:Mg quantum well, and ~10 nm GaN:Mg. The Al and In compositions in the active region can be varied by adjusting the Al/In flux ratios and/or the growth temperatures to control the emission wavelengths of these AlInN UV nanowire LEDs. As illustrated in Fig. 2(b), the nanowires are perpendicularly arranged on the substrate and exhibit quite uniform heights, with diameters at the top of the nanowire in the range of ~90 nm. Such nanowire properties are suitable for device fabrication.

### Optical characterizations

**Figure 3:**
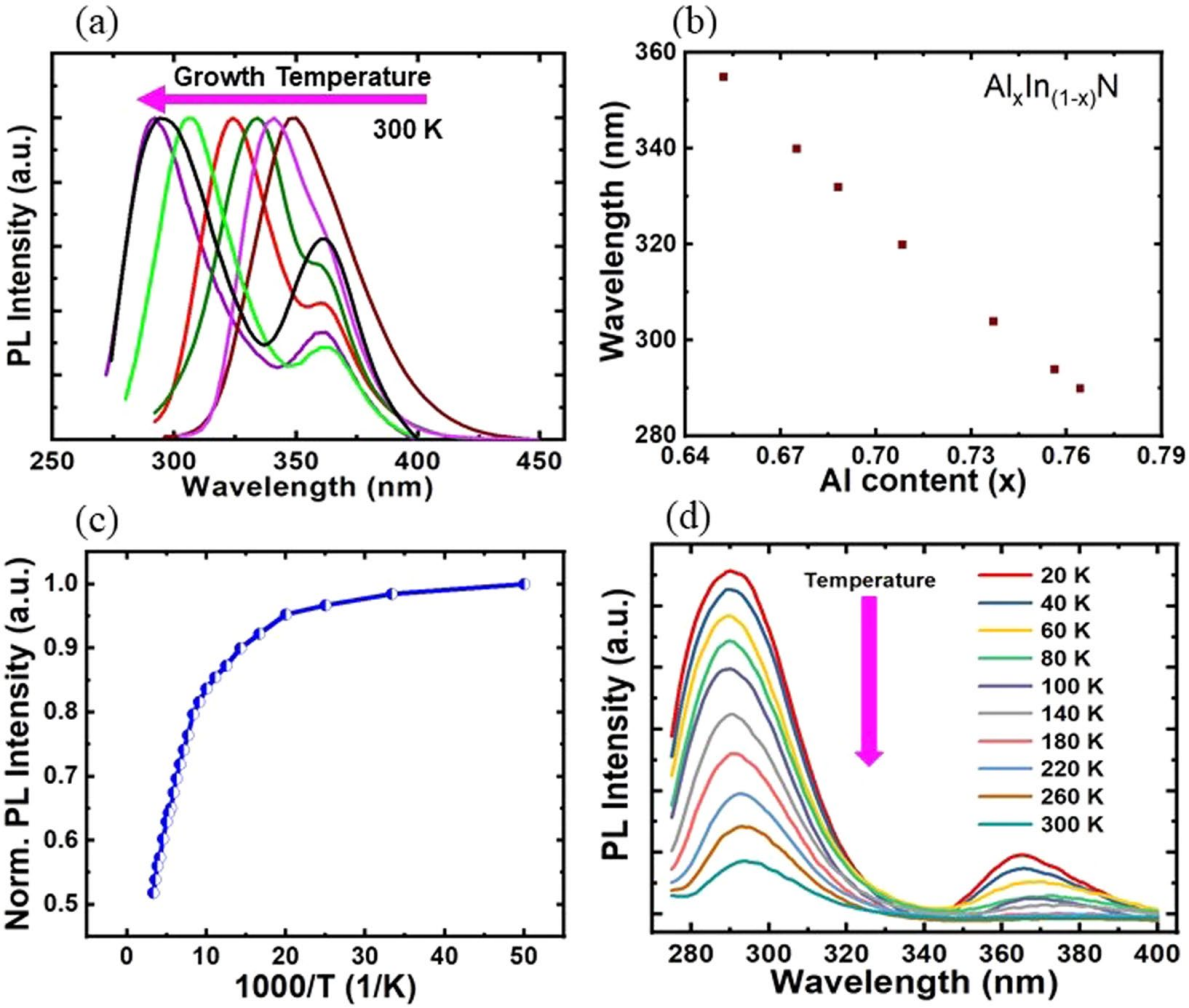
(**a**) Photoluminescence spectra of AlInN/GaN nanowires. The peak emission varied from 290 nm to 355 nm. (**b**) Photoluminescence peak wavelength versus estimated Al composition. (**c**) Temperature-dependent photoluminescence intensity of AlInN/GaN nanowires measured from 20 K to 300 K. (**d**) Photoluminescence spectra of AlInN/GaN nanowires measured from 20 K to 300 K under an excitation power of 10 mW.

The photoluminescence (PL) spectra of AlInN nanowires on GaN templates were characterized using a 266 nm laser. Figure 3(a) presents the PL spectra of different AlInN/GaN nanowire structures grown under different growth conditions. It is clearly shown that the peak emissions varied from 290 nm to 355 nm by varying the Al composition in the AlInN layers. In this study, the Al/In beam equivalent pressure (BEP) ratio was kept constant while the substrate temperature was increased from 670 °C to 720 °C. The nitrogen flow rate was maintained at 2.5 sccm. The peak emission shifted to shorter wavelengths when the substrate temperature increased, which is attributed to the increased In adatom desorption at higher growth temperatures, resulting in a reduced In composition in the AlInN segment. As illustrated in Fig. 3(a), the peak wavelength at ~368 nm is related to the emission from GaN nanowire templates. We also estimated the Al composition in the AlInN layer using the following equation:

E_Peak_(*x*) ≈ E_g_(*x*) = *x*E_g_(AlN) + (1-*x*)E_g_(InN) − b*x*(1 − *x*), where E_Peak_ is the PL peak energy at room temperature, *x* is the Al composition, and Eg is the bandgap energy. In our calculation, E_g_(AlN) and E_g_(InN) were determined to be 6.2 eV^45^ and 0.64 eV^45^, respectively, and b was taken as the bowing parameter. b was chosen to be 3.4 eV^46^. As shown in Fig. 3(b), the Al content was estimated to be in the range of 65.1–76.4%, corresponding to an emission wavelength from 290 nm to 355 nm. At a growth temperature of 710 °C, AlInN nanowires with an emission wavelength of 295 nm were recorded with a strong emission intensity and a spectral linewidth of ~28 nm. The optical properties of those AlInN/GaN nanowires were further characterized at different temperatures varying from 20 K to 300 K using liquid helium under an excitation power of 10 mW to estimate their IQEs. The IQE was calculated by comparing the integrated PL intensities of emission from the AlInN layer at room temperature and at 20 K, assuming the IQE at 20 K is near unity^47^. As presented in Fig. 3(c), the AlInN/GaN nanowire exhibited a relatively high IQE, which was estimated to be ~52% at room temperature, attributed to the strong carrier confinement provided by the AlInN shell and nearly intrinsic AlInN core. Figure 3(d) presents the temperature-dependent PL spectra of AlInN/GaN nanowires measured from 20 K to 300 K. At 20 K, strong emissions at ~290 nm and ~364 nm were recorded for emissions from AlInN and GaN segments, respectively. S-shaped behaviour was not observed for the peak position of emission from the AlInN segment. At room temperature, the peak emission at 295 nm was from the AlGaN segment, while the peak emission at ~368 nm was related to the emission from the GaN layer. When the temperature increased from 20 K to 300 K, redshifts were observed for peak wavelength emission from AlGaN and GaN, respectively. The redshifts may be due to the bandgap shrinkages of AlGaN and GaN when the temperature increases^44^.

### Device performance

**Figure 4:**
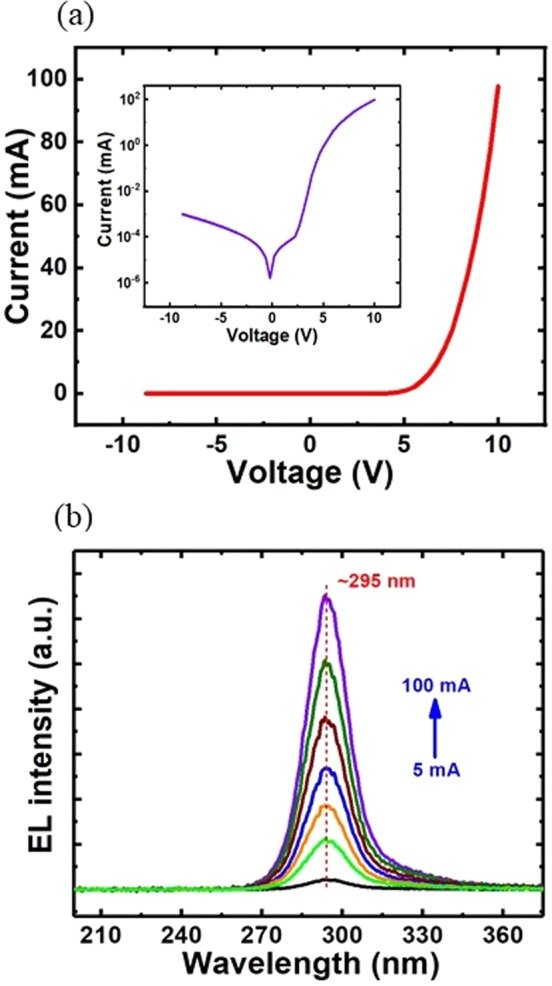
(**a**) I-V characteristics of the AlInN UV nanowire LED. The inset shows the I-V characteristics of the AlInN UV LED device on a semi-log scale. (**b**) Electroluminescence (EL) spectra of the AlInN UV nanowire LEDs under an injection current range of 5–100 mA.

Such vertically aligned AlInN nanowire LEDs are fully compatible with the conventional fabrication process for large-area nanowire LED devices. The device fabrication is described in the method section. UV nanowire LED devices with an areal size of 500 × 500 µm^2^ were chosen for characterization. The AlInN LEDs have excellent current-voltage characteristics with low resistance measured at room temperature, as shown in Fig. 4(a). The leakage current was found to be very small, with a value of approximately 1 µA at −8 V. The turn-on voltage of these UV nanowire LEDs was ~5 V, which is significantly lower than that of the current thin-film AlGaN LEDs in similar wavelength range^48,49^ and is also better than/comparable to that of currently reported AlGaN UV nanowire LEDs^43,47,50^. Figure 4(b) presents the electroluminescence (EL) spectra of the AlInN nanowire LEDs under various injection currents from 5 mA to 100 mA. No obvious shift in the peak wavelength was observed, attributed to the negligible quantum-confined Stark effect (QCSE) in the LED structures, further confirming the high crystalline quality of such AlInN nanowire heterostructures.

**Figure 5:**
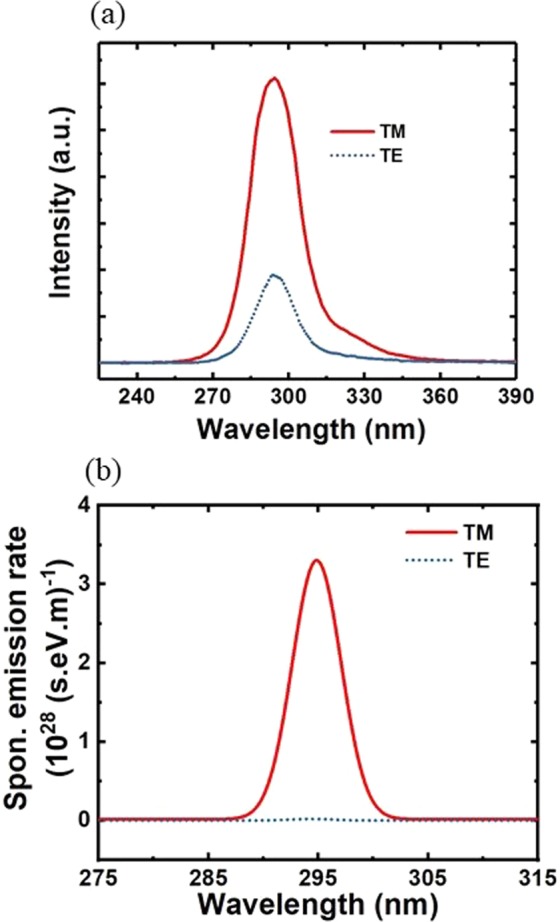
(**a**) TM and TE polarized spectra of the AlInN UV nanowire LED with an emission wavelength of 295 nm measured at 10 A/cm^2^. (**b**) The simulation results of TM- and TE-polarized emission from AlInN nanowire LEDs at 295 nm.

The light emission polarization properties of the AlInN UV nanowire LEDs were also characterized at room temperature. Transverse-magnetic (TM) and transverse-electric (TE) are defined as the electric field parallel (**E**//**c**) and perpendicular (**E** ± **c**) to the c-axis, respectively. The measurement was performed at an injection current of 10 A/cm^2^. As illustrated in Fig. 5(a), the UV light emission was predominantly TM polarized, which is approximately >4 times stronger than that of TE polarized emission. This observation agrees well with the simulation results in which the TM-polarized emission was approximately two orders of magnitude stronger than TE-polarized light, as shown in Fig. 5(b). A similar trend of polarization for LEDs using AlGaN in the same UV wavelength regime has also been reported by others^43,51^. This result plays an important role in the design of surface-emitting UV LEDs using AlInN compounds to achieve high light extraction efficiency.

## Discussion

**Figure 6:**
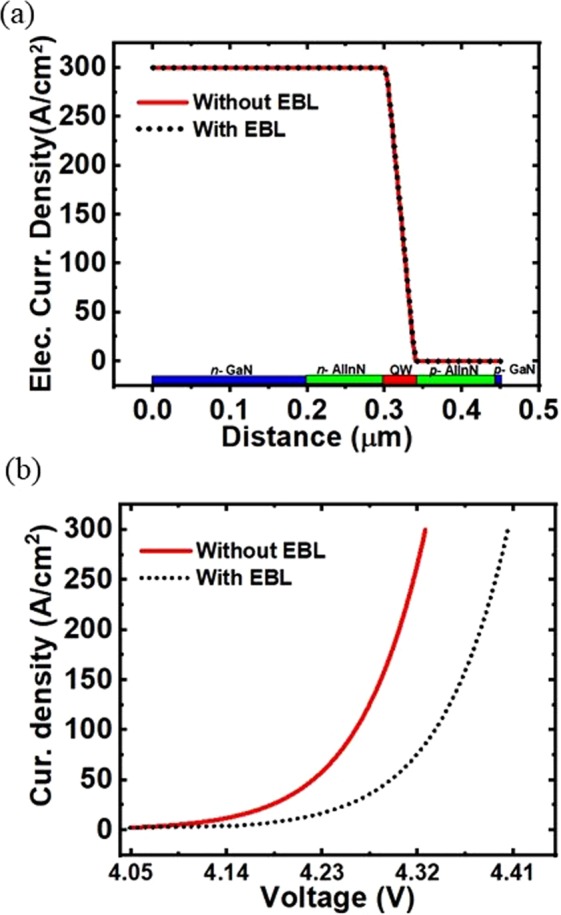
(**a**) The simulated electron current density of the AlInN nanowire LEDs with and without EBL showing a similar trend of electron current distribution. (**b**) The simulated I-V characteristics of AlInN nanowire LEDs with and without EBL.

In addition to studying the performance of AlInN UV nanowire LEDs, we performed detailed simulations comparing the characteristics of AlInN nanowire LEDs with and without the integration of an electron blocking layer. It is clearly shown that in both LED device structures, electron leakage does not occur or is negligible with a similar electron current density distribution, as shown in Fig. 6(a). However, EBL has a strong impact on hole injection efficiency. The EBL-free LED has better hole injection efficiency than other LEDs. It is observed in the band diagram of the LED with EBL that there is band bending in the valence band at the heterointerface of the EBL and quantum well (see Fig. S1(a) in the Supporting Information), resulting in hole accumulation at the starting portion of the EBL (see Fig. S1(b) in the Supporting Information). This phenomenon decreases the hole injection efficiency in the quantum well and leads to a higher turn-on voltage for AlInN UV nanowire LEDs with EBL, as shown in Fig. 6(b). The advantages of AlInN nanowire structures include the integration of such nanowire UV LED structures on GaN templates as well as a simple structure without the use of an EBL for high device performance. The EBL-free LED structure is particularly important for developing deep UV LEDs since the Al composition almost reaches a maximum for deep-UV emission (below 240 nm). Therefore, the optimal EBL structure is limited, which requires a higher bandgap energy to effectively prevent electron overflow. Moreover, the use of EBL will also affect hole transport, resulting in reduced hole injection efficiency to the device active region. Further optimization of the device structure, active region thickness and composition will be performed to achieve high-power AlInN deep-UV nanowire LEDs.

In summary, we have successfully demonstrated the first AlInN axial nanowire LEDs operating in the UV-A and UV-B bands with a relatively high IQE of ~52% at room temperature. Electron overflow was not observed within these nanowire UV emitters. The devices exhibit stable emission with strong TM-polarized light emission. The device performance can be further improved by engineering the device structure, nanowire morphology and nanowire diameter and spacing to enhance the light extraction efficiency of such AlInN UV core-shell nanowire LEDs.

## Materials and Methods

### Molecular beam epitaxial (MBE) growth

Vertically aligned self-organized AlInN/GaN heterostructures and AlInN core-shell nanowire LEDs were grown on Si(111) substrates by radio-frequency plasma-assisted molecular beam epitaxy. An extremely high-purity nitrogen generation system was employed to introduce ultrahigh-quality nitrogen gas to the RIBER RF-nitrogen plasma cell. This system includes a Delux nitrogen purifying system with a bypass assembly life status indicator, valve control for bypass and a purifier and heating control. The oxide on the substrate surface was desorbed *in situ* at 780 °C. First, GaN nanowire templates were formed under nitrogen-rich conditions without the use of any external catalyst. The growth conditions of the GaN nanowires included a growth temperature of 770 °C, a nitrogen flow rate of 1.0 sccm, a forward plasma power of 400 W, and a Ga beam equivalent pressure of 6 × 10^−8^ Torr. To achieve UV light emission, self-organized AlInN segments were subsequently grown on top of the GaN nanowires. The In composition in the active region could be controlled by varying the In and Al beam flux and/or the substrate temperature. The growth temperature of the AlInN active regions was varied to enhance the In incorporation, which was controlled between 670 °C and 720 °C. During the epitaxial growth of AlInN segments, the nitrogen flow rate and plasma power were maintained at 2.5 sccm and 400 W, respectively.

### Fabrication process

The UV nanowire LED fabrication process includes the following steps. The nanowire LED samples were first cleaned by HCl and then HF to remove native oxides from the nanowire surface and the oxide layer from the backside of the Si substrates. Ti(20 nm)/Au(120 nm) metal layers were then deposited on the backside of Si wafers for *n*-contact. The *p*-metal contact of Ni(10 nm)/Au(10 nm) was deposited on top of the nanowire samples by e-beam evaporation. To enhance the efficient current spreading of this *p*-contact layer, the top portions of nanowires must be linked together, which can be achieved by tilting the substrate holder at a certain angle during deposition. Thick Ni(20 nm)/Au(120 nm) layers were subsequently deposited on top of the device to serve as a metal pad. The fabricated devices with Ti/Au and Ni/Au contacts were annealed at ~550 °C for 1 min. Filling materials and indium tin oxide (ITO) were not used in this fabrication to eliminate any light absorption in this UV wavelength range, which is different from our visible color InGaN/(Al)GaN nanowire LED fabrication. LEDs with chip areas of ~500 × 500 µm^2^ were fabricated and selected for characterization.

### Transmission electron microscopy (TEM)

A JEOL JEM-2100F equipped with a near-field emission gun with an accelerating voltage of 200 kV was used to obtain bright-field TEM images. For STEM-and STEM-HAADF imaging, the same equipment with a cold field emission emitter operated at 200 kV and an electron beam diameter of approximately 0.1 nm was used.

### Photoluminescence measurement

A 266 laser (Kimmon Koha) was used as the excitation source for the photoluminescence measurement of the nanowire heterostructure. The photoluminescence was spectrally resolved by a high-resolution spectrometer and detected by a photomultiplier tube (PMT).

### Electrical, electroluminescence and light polarization characterization

The current-voltage characteristics of AlInN UV core-shell nanowire LEDs were measured by means of a Keithley 2400 digital source meter. The electroluminescence emission of the LED devices was collected by an optical fibre and analysed using an Ocean Optics spectrometer. The light emission polarization set up consisted of an optical fibre together with a Glan-Taylor polarizer mounted on a rotating arm. Signals from the lateral surface of AlInN nanowire LEDs were polarization resolved by a polarizer and collected and analysed by means of an Ocean optics spectrometer. The measurement was performed at a 20 mA CW injection current.

## Supplementary information

SUPPLEMENTARY INFORMATION.
