# Supplementary material for: Epitaxial Growth and Characterization of AlInN-Based Core-Shell Nanowire Light Emitting Diodes Operating in the Ultraviolet Spectrum
Source: Sci Rep. 2020 Feb 13;10:2547. doi: 10.1038/s41598-020-59442-0 (PMC7018839; doi:10.1038/s41598-020-59442-0)
Supplement: Supplementary file 1 — SUPPLEMENTARY INFORMATION. [file 41598_2020_59442_MOESM1_ESM.pdf]

Supplementary Information

Epitaxial Growth and Characterization of AlInN Based Core-Shell Nanowire Light Emitting Diodes Operating in the Ultraviolet Spectrum

Ravi Teja Velpula[1], Barsha Jain[1], Moab Rajan Philip[1], Hoang Duy Nguyen[2,¥], Renjie Wang[3], and Hieu Pham Trung Nguyen[1,*]

[1]*Department of Electrical and Computer Engineering, New Jersey Institute of Technology, 323 Dr Martin Luther King Jr Boulevard, Newark, New Jersey, 07102*

[2]*Institute of Chemical Technology, Vietnam Academy of Science and Technology, 1 Mac Dinh Chi Street, District 1, Ho Chi Minh City 700000 Vietnam*

[3]*Department of Engineering Physics, McMaster University, 1280 Main Street West, Hamilton, Ontario, L8S 4L7 Canada*

E-mail: [*]hieu.p.nguyen@njit.edu; [¥]nhduy@iams.vast.vn

S1. Device Modeling

The optical and electrical properties of AlInN based deep ultraviolet (DUV) light-emitting diodes (LEDs) are modeled and calculated using Crosslight Advanced Physical Models of Semiconductor Devices (APSYS) 2018 simulation software. These properties include electron concentration, hole concentration, electron current density, hole current density, electrostatic field, radiative recombination rate, TE/TM radiative recombination rates, internal quantum efficiency (IQE), I-V, and L-I. The APSYS simulation package uses 2D/3D finite element analysis of electrical, optical and thermal properties of silicon and compound semiconductor devices and solves the poison's equation, the current continuity equation, the carrier energy transport equation, quantum

mechanical wave equations and the scalar wave equations for the photonic devices. A comparison of device performance between AlInN nanowire LED with and without the presence of an electron blocking layer (EBL) is performed and analyzed. The EBL has been employed in the LED structure to reduce or eliminate the electron overflow outside of the LED active region[1-4], therefore, the efficiency droop was reduced. However, for deep UV LEDs based on III-nitride semiconductor, the Al composition of the active region is almost reaching maximum of more than 80%, the design and employment of an efficient EBL are difficult since it required having its bandgap energy larger than that of the active region. Such large bandgap EBL segment adversely affects the hole transport and injection into the active region, leading to highly nonuniform carrier distribution[3,5-7]. In this study, we report that AlInN nanowire LEDs offer uniform carrier distribution, leading to an efficient EBL-free LED structure which exhibits better performance compared to LEDs using an EBL. The device active region consists of 200 nm *n*-GaN template, a single quantum well (QW) of 40 nm $Al_{0.841}In_{0.159}N$ active region, sandwiched by 100 nm *n*-$Al_{0.865}In_{0.135}N$ and 100 nm *p*-$Al_{0.865}In_{0.135}N$, and finally a 10 nm *p*-GaN contact layer. For AlInN LED with an EBL, a 10 nm *p*-$Al_{0.875}In_{0.125}N$ is considered as an EBL and placed after the quantum well.

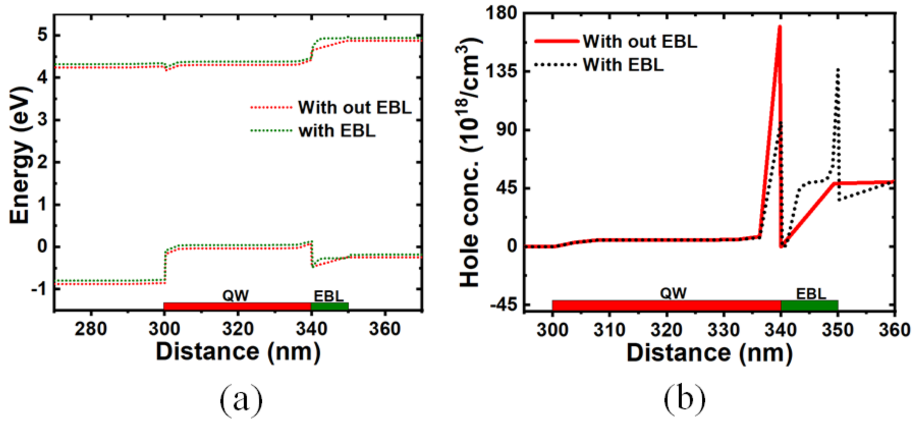

Figure S1: (a) Band diagrams of LEDs with and without EBL. (b) Hole concentration in LED with and without EBL.

In the case of LED with the EBL, band bending can be observed as a result of polarization mismatch in the valence band at the heterointerface of the EBL and quantum well and is shown in the Figure S1(a). As a result of this, holes are accumulating in the starting portion of the EBL. This phenomena decreases the hole injection efficiency in the quantum well and is shown in the Figure S1(b). The EBL free LED has better hole injection efficiency compared to the other.

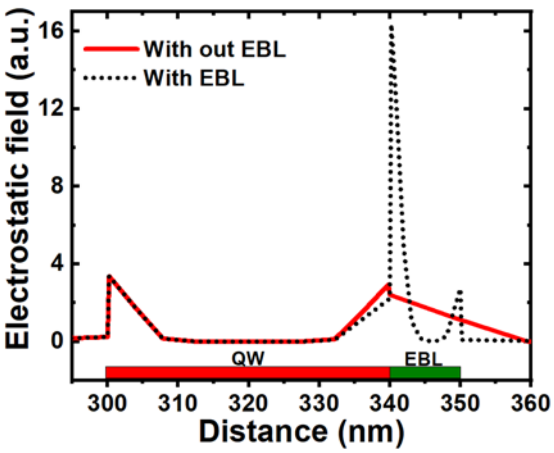

Figure S2: Electrostatic field distributions in AlInN nanowire LEDs with and without EBL.

Figure S2 shows the electroststic field in the quantum wells of the both LED devices. It is clearly shown that both the devices have almost same electrostatic field in quantum well but LED with EBL has more electric field at the heterointerface of the quantum well and EBL due to more lattice mismatch. This decreses the hole injection efficiency in the device with EBL. The EBL free LED has lower turn-on voltage compared to the other device because it has better hole injection effciency.

We have further analyzed the emission light polarization in EBL-free AlInN nanowire LED. Figures S3(a) and S3(b) present the TE and TM spontaneous emission rates of AlInN UV nanowire LED. It is clearly shown that TM radiative recombination is significantly dominant in the UV region which is about 2 orders of magnitude higher than TE radiative recombination.

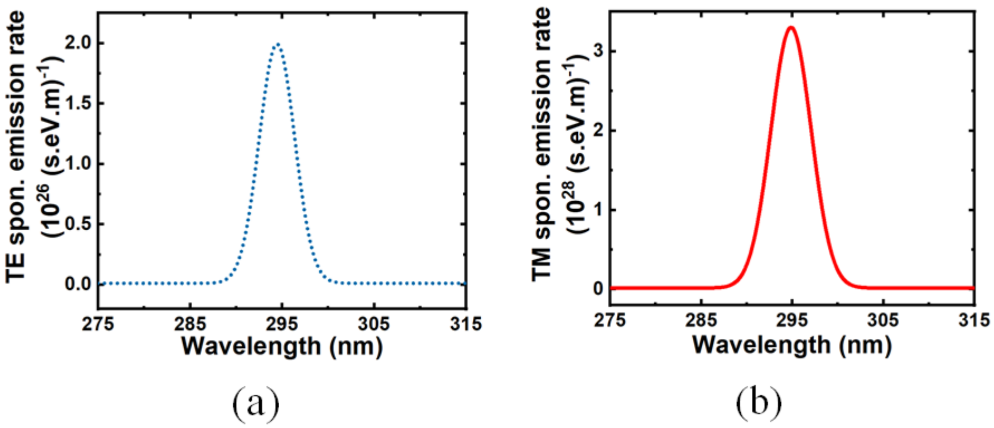

Figure S3: (a) TE spontenous emission rate and (b) TM sponstanous emission rate from AlInN UV nanowire.

The EBL-free LED structure is particularly important for developing deep UV LEDs with emission wavelength below 240 nm since the Al composition almost reaches maximum in this region. Therefore, the optimal EBL structure is limitted which requires higher bandgap energy to effectively prevent electron overflow. Moreover, the use of EBL will also affect the hole transport, resulted to the reduced hole injection efficiency to the device active region [8,9]. As a part of this analysis, it is necessary to understand the role of the EBL in DUV LEDs. To understand this, we have simulated three different LED structures named as LED1, LED2 and LED3 with emission wavelength around 236 nm. LED1 contains 200 nm *n*-GaN template, 100 nm *n*-$Al_{0.92}Ga_{0.08}N$, active region consists of 3 nm *i*-$Al_{0.85}Ga_{0.15}N$ QW which is sandwiched between 3nm *i*-$Al_{0.92}Ga_{0.08}N$ quantum barriers (QB), 100 nm *p*-$Al_{0.92}Ga_{0.08}N$ and 10 nm *p*-GaN contact layer. LED2 is same as LED1, except 10nm *p*-$Al_{0.97}Ga_{0.03}N$ EBL after the active region in the device. LED3 consists of 200 nm *n*-GaN template, 3 nm QB (*i*-$Al_{0.98}In_{0.02}N$)/QW (*i*-$Al_{0.955}In_{0.045}N$)/QB (*i*-$Al_{0.98}In_{0.02}N$) is sandwiched by 100 nm *n*-$Al_{0.98}In_{0.02}N$ and *p*-$Al_{0.98}In_{0.02}N$ and a 10 nm *p*-GaN contact layer.

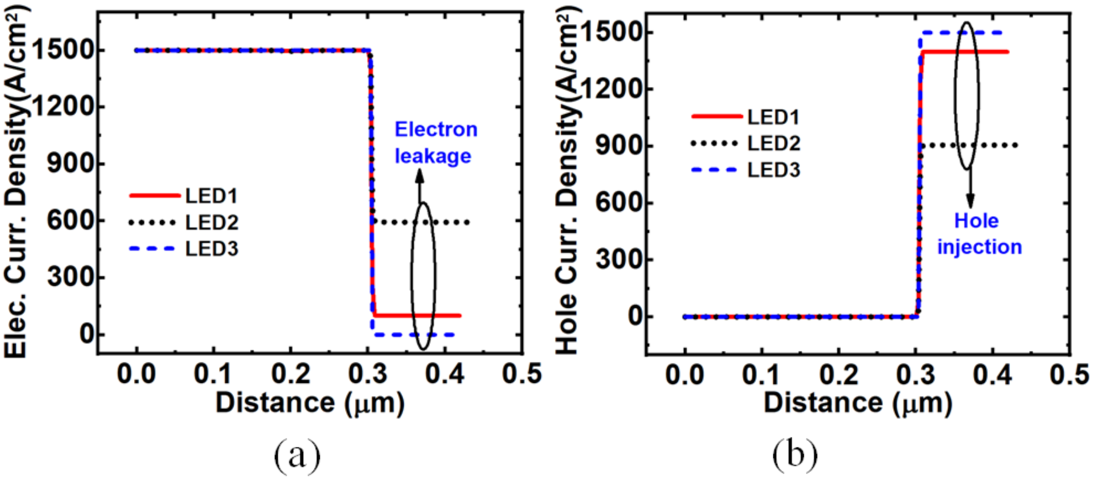

Figure S4: (a) Electron current density and (b) Hole current density of LED1, LED2 and LED3 at 236 nm emission wavelength.

Figure S4 shows the electron and hole current density of LED1, LED2 and LED3. The integration of EBL in AlGaN LEDs leads to have more electron leakage from the active region and can be understood from Figure S4 (a). This is due to poor hole injection into the active region and can be seen from Figure S4 (b). At this wavelenght, integration of EBL deteriorates the device performance, hence, EBL free LEDs with negligible electron leakage are necessary. EBL free AlInN LED which is LED3 has negligible electron leakage from the active region and better hole injection into the active region compared to LED1 and LED2. Hence EBL free AlInN based LEDs are proming candidate for deep UV region applications.

S2. Electroluminescence Characterization

Figure S5 shows the normalized electroluminescence spectra of AlInN based UV core-shell nanowire LEDs. The peak emission varies from ~290 nm to ~ 355 nm which is agreed well with the PL results shown in Figure 3(a).

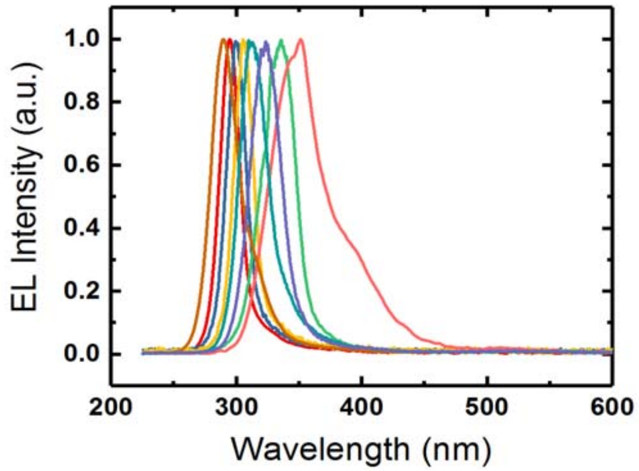

Figure S5: Electroluminescence spectra of different AlInN based UV nanowire LEDs grown at different conditions.

References:

1 Özgür, Ü. *et al.* Ballistic transport in InGaN-based LEDs: impact on efficiency. *Semiconductor Science and Technology* **26**, 014022 (2011).

2 Nguyen, H. P. T. *et al.* Controlling Electron Overflow in Phosphor-Free InGaN/GaN Nanowire White Light-Emitting Diodes. *Nano Letters* **12**, 1317–1323 (2012).

3 Han, S.-H. *et al.* Effect of electron blocking layer on efficiency droop in InGaN/GaN multiple quantum well light-emitting diodes. *Applied Physics Letters* **94**, 231123 (2009).

4 Singh, S., Robidas, D., Rohila, N., Pal, S. & Dhanavantri, C. Effect of electron blocking layer on efficiency droop in blue InGaN/GaN based light-emitting diodes. *Optoelectronics and Advanced Materials-Rapid Communications* **4**, 1106-1110 (2010).

5 Xia, C. S. *et al.* Efficiency enhancement of blue InGaN/GaN light-emitting diodes with an AlGaN-GaN-AlGaN electron blocking layer. *Journal of Applied Physics* **111**, 094503 (2012).

6 Kim, M. H. *et al.* Origin of efficiency droop in GaN-based light-emitting diodes. *Applied Physics Letters* **91**, 183507 (2007).

7 Wang, C. H. *et al.* Hole injection and efficiency droop improvement in InGaN/GaN light-emitting diodes by band-engineered electron blocking layer. *Applied Physics Letters* **97**, 261103 (2010).

8 Wang, N., Yin, Y. A., Zhao, B. & Mei, T. Performance Analysis of GaN-Based Light-Emitting Diodes With Lattice-Matched InGaN/AlInN/InGaN Quantum-Well Barriers. *Journal of Display Technology* **11**, 1056-1060 (2015).

9 Choi, S. *et al.* Efficiency droop due to electron spill-over and limited hole injection in III-nitride visible light-emitting diodes employing lattice-matched InAlN electron blocking layers. *Applied Physics Letters* **101**, 161110 (2012).
